# Epigenetic regulation and beyond in grapevine‐pathogen interactions: a biotechnological perspective

**DOI:** 10.1111/ppl.70216

**Published:** 2025-04-15

**Authors:** João Proença Pereira, Ivan Bevilacqua, Rita B. Santos, Serena Varotto, Walter Chitarra, Luca Nerva, Andreia Figueiredo

**Affiliations:** ^1^ Grapevine Pathogen Systems Lab BioISI – Biosystems & Integrative Sciences Institute (BioISI), Faculty of Sciences, University of Lisbon Lisboa Portugal; ^2^ Department of Agronomy Food Natural Resources, Animals and Environment (DAFNAE) University of Padova Legnaro (PD) Italy; ^3^ Council for Agricultural Research and Economics—Research Centre for Viticulture and Enology (CREA‐VE) Conegliano (TV) Italy; ^4^ Plant Biology Department, Faculty of Sciences BioISI, University of Lisbon Lisboa Portugal

## Abstract

As one of the most important crop plants worldwide, understanding the mechanisms underlying grapevine response to pathogen attacks is key to achieving a productive and sustainable viticulture. Recently, epigenetic regulation in plant immunity has gained significant traction in the scientific community, not only for its role in gene expression regulation but also for its heritability, giving it enormous biotechnological potential. Epigenetic marks have been shown to be dynamically modulated in key genomic regions upon infection, with some being maintained after such, being responsible for priming defense genes. In grapevine, however, knowledge of epigenetic mechanisms is still limited, especially regarding biotic stress responses, representing a glaring gap in knowledge in this important crop plant. Here, we report and integrate current knowledge on grapevine epigenetic regulation as well as non‐epigenetic non‐coding RNAs in the response to biotic stress. We also explore how epigenetic marks may be useful in grapevine breeding for resistance, considering different approaches, from uncovering and exploiting natural variation to inducing it through different means.

## 1 INTRODUCTION

The current agricultural landscape sits at a crossroads. While securing productivity is crucial to meeting the ever‐increasing societal demands, agriculture is facing threats from various fronts, which jeopardize it. Among them, diseases are one of the most significant factors that come to compromise both productivity and sustainability as they are fought with the extensive use of environmentally deleterious phytopharmaceuticals. Because of this, the search for new mechanisms and methodologies that aim to increase plant resilience towards disease has become increasingly important, enabling the development of more tolerant crops to mitigate the use of these products.

Epigenetic regulation consists of modifications occurring at the DNA level that alter gene function without altering the underlying genomic sequence, and that are also inherited both mitotically and meiotically (Deans and Maggert 2015). These modifications, such as DNA methylation or histone modifications, play a key role in transposable element (TE) silencing and gene expression regulation, among other processes (Pikaard and Mittelsten Scheid 2014). Considering the two main features of epigenetic regulation – gene expression control and heritability ‐ it is no surprise that it has garnered significant attention in the context of plant‐pathogen interactions. On the one hand, transcriptional reprogramming is one of the hallmarks of the early stages of an infection, often dictating the outcome of the interaction (Birkenbihl et al. 2017). On the other hand, memory of infection at different timescales is of relevance to the fate of subsequent attacks, whether it be in the same generation (i.e., somatic memory), the next one (i.e., intergenerational memory) or many generations after the initial exposure (i.e., transgenerational memory) (Mladenov et al. 2021; Hannan Parker et al. 2022; Gallusci et al. 2023). When considering biotechnological approaches, inter‐ and transgenerational memory are essential for either annual plants or clonally propagated crops if the breeding process involves sexual reproduction. Conversely, long‐term somatic memory in clonally propagated crops is of greater importance, although it was recently argued that this form of propagation is not as conducive to maintaining a memory state (Ibañez and Quadrana 2023).

Grapevine (*Vitis vinifera* L.) is renowned for its quality and finds extensive application in wine production. One of the greatest threats to yields in viticulture are its common diseases, which are fought with the extensive use of phytopharmaceuticals, representing one of the main contributing factors to the unsustainability of this sector (Fouillet et al. 2022). It is therefore worthwhile to better understand how grapevine responds to common pathogens at the molecular and genetic levels, with epigenetics posing an interesting study target for this very aim. Indeed, grapevine has emerged as a promising woody crop plant model for studying epigenetic processes due to the availability of a reference genome rich in TEs, which are more prone to silencing via DNA methylation (see section Mechanisms of epigenetic regulation). Considering this high percentage of TEs (*circa* 40% of the genome; The French–Italian Public Consortium for Grapevine Genome Characterization 2007), one would expect that DNA methylation modulation would be of greater significance in grapevine than in plants with lower amounts of TEs like Arabidopsis (circa 14% of the genome; The Arabidopsis Genome Initiative 2000).

Although still in its infancy, epigenetic research in grapevine is gaining traction in the scientific community. In fact, epigenetic regulation and its potential for improving the response of grapevine to stress has recently been discussed both in the scope of climate change (Berger et al. 2023; Tan and Rodríguez López 2023) and in its agronomical potential as a whole (Venios et al. 2024). However, the potential role of epigenetics in enhancing grapevine resilience to diseases remains unclear and has not been addressed within the scope of the current research. In this review, we aim to discuss the prospect of epigenetics in grapevine breeding for increased tolerance to diseases. We summarize current knowledge on epigenetic regulation in grapevine‐pathogen interactions, as well as other non‐epigenetic regulatory mechanisms, and speculate on ways these mechanisms can be harnessed in breeding, along with their feasibility in grapevine.

## 2 MECHANISMS OF EPIGENETIC REGULATION

**FIGURE 1:**
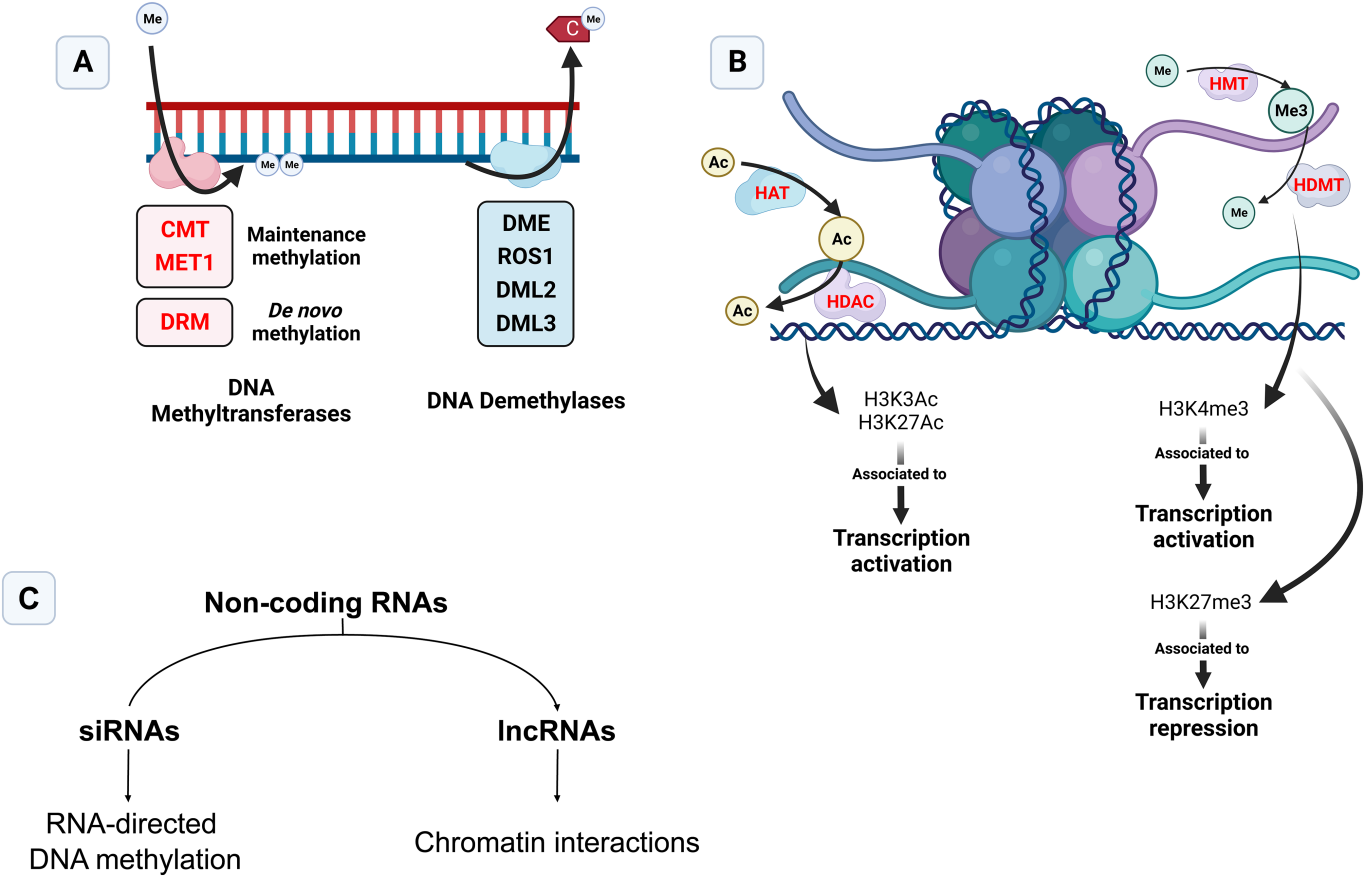
Main mechanisms of epigenetic regulation. A – mechanisms of DNA methylation; B – mechanisms of histone modifications; C – mechanisms of epigenetic regulatory ncRNA and their functions. Text in red represents gene families and functions already identified in grapevine. CMT – Chromomethylase; MET1 – Methyltransferase 1; DRM – Domains rearranged methyltransferase; DME – Demeter; ROS1 – Repressor of silencing 1; DML2 – Demeter‐like 2; DML3 – Demeter‐like 3; HAT – Histone Acetyltransferase; HDAC – Histone Deacetylase; HMT – histone methyltransferase; HDMT – Histone demethylase; siRNA – small interfering RNA; lncRNA – long non‐coding RNA; Ac – acetyl group; Me – methyl group. Created with BioRender.

Different epigenetic mechanisms and pathways contribute to epigenetic state stability and modification in plant cells and tissues during development and in response to the environment. Epigenetic states are characterized by different combinations of epigenetic marks, which include DNA methylation, histone post‐translational modifications (HPTM) and regulation by regulatory non‐coding RNAs (ncRNAs) like small interfering RNA (siRNA) and long non‐coding RNA (lncRNA) (Figure 1).

In plants, DNA methylation status is mediated by two enzyme families, DNA methyltransferases and DNA demethylases, which catalyze the addition of a methyl group to cytosines and excision of 5‐methylcytosine (5mC) from DNA, respectively. Unlike mammals, DNA methylation in plants occurs in three distinct sequence contexts: CG, CHG and CHH (H = A, T, G). DNA methyltransferases can be divided into 3 distinct classes: methyltransferase 1 (MET1), chromomethylases (CMT), and domains rearranged methyltransferase (DRM) (Zhang et al. 2018), with each class playing a specific role. For instance, CMT and MET1 are linked to maintenance methylation (i.e., assuring full methylation of hemi‐methylated sites), MET1 is linked to CG maintenance, while CMT3 and CMT2, albeit to a lesser extent, are linked to CHG and CMT2 and DRM to CHH in a siRNA‐dependent mechanism (Zhang et al. 2018). Furthermore, *de novo* DNA methylation, assured by DRM2, is directed by siRNAs loaded into ARGONAUTE4/6 (AGO4/6) in the RNA‐directed DNA methylation (Erdmann and Picard 2020). Active DNA demethylation is mediated by DNA demethylases, namely DEMETER (DME), REPRESSOR OF SILENCING 1 (ROS1), DME‐like 2 (DML2) and DME‐like 3 (DML3), which, unlike DNA methyltransferases, appear to not have an affinity for particular sequence contexts (Parrilla‐Doblas et al. 2019).

Methylation occurs in various genomic regions, achieving different biological functions depending on where it is present. A canonical function for DNA methylation is the repression of TEs by both blocking their movement and inhibiting their insertion in other regions, contributing to genomic stability. As for its role in gene expression, it appears to be more complex. For instance, promoter methylation is widely associated with transcriptional repression due to either inhibition of transcription factor binding or the binding of methylation‐associated transcriptional repressors (O'Malley et al. 2016; Charvin et al. 2023). Interestingly. the reverse case has been described for *ROS1* expression in *Arabidopsis* (Lei et al. 2015; Williams et al. 2015) and in tomato ripening‐associated genes (Lang et al. 2017), suggesting other mechanisms could interplay with DNA methylation. The role of intragenic methylation is also not fully known, with possible different roles ascribed to where inside the gene it is located. For example, exon methylation (known as gene body methylation, gbM), initially thought to be a positive regulator of gene expression, is hypothesized to play many more roles, from preventing aberrant transcription to regulating splicing. Furthermore, a role in protecting the coding regions of genes from unwanted TE insertion has also been put forth (reviewed in Muyle et al. 2022). To date, in grapevine, only DNA methyltransferases have been characterized at both the bioinformatic and expression level, in the context of infection with downy mildew (Pereira et al. 2022). DNA demethylases have been studied in the context of fruit ripening (Shangguan et al. 2020), but have not been characterized in terms of pathogen infection.

Regarding the role of histones in epigenetic regulation, it is important to consider both the role of histone variants and their post‐translational modifications. The nucleosome is composed of four histone types (H2A, H2B, H3 and H4) consisting of a core (H3‐H4)2 tetramer and two H2A‐H2B dimers (Luger et al. 1997). The amino acid tails of these histones can be modified by writers and erasers of histone marks, impacting chromatin accessibility and gene expression states. Perhaps the most studied modifications are histone methylation and acetylation. Histone methylation is added by the writers Histone methyltransferases (HMT) and removed by the erasers Histone demethylases (HDMT) (Hu and Du 2022). Depending on the methylated amino acid residue and genomic location, different modifications can play different roles. For instance, the marks H3K4me2/3, generally deposited in gene promoters, are associated with opposite roles in gene expression regulation, with dimethylation linked to repression and trimethylation associated with activation. In addition, marks such as H3K9me2 and H3K4me1 have been linked to crosstalk with DNA methylation, the former being involved in CMT2/3‐mediated non‐CG methylation, and the latter in the recruitment of RdDM machinery (Niu et al. 2021; Hu and Du 2022). Acetylation, on the other hand, is added by Histone acetyltransferases (HAT) and removed by histone deacetylases (HDAC). As opposed to methylation, histone acetylation is typically associated with transcriptionally active chromatin, due to the disruption of the DNA‐histone interaction by the acetyl group (Kumar et al. 2021). Additionally, nonallelic sequence variants of canonical histones that have introns exist in plant genomes, particularly of H2A and H3, such as H2A.Z or H3.1, respectively, and that can be incorporated into nucleosomes in a targeted and replication‐independent manner. Due to their sequence divergence from canonical histones, histone variants give specific properties to the nucleosomes they occupy and can affect epigenetic states, transcription and genome stability (Foroozani et al. 2022). Perhaps the best‐characterized histone variant in the context of biotic stress is H2A.Z, which is predominantly deposited by the SWR1‐C complex in the nucleosomes of environmentally responsive genes (ERGs). Indeed, loss of function of H2A.Z, as well as components of the SWR1‐C complex, were noted to impair effector‐triggered immunity (ETI), as well as salicylic acid (SA)‐ and jasmonic acid (JA)‐associated hormonal signalling (Berriri et al. 2016). Furthermore, H2A.Z may also play a key role in the priming of defense genes, interplaying with RdDM‐related mechanisms (Hannan Parker et al. 2022) (see Epigenetics and plant immunity – another layer of regulation).

In grapevine, both HATs and HDACs have been characterized bioinformatically and in the expression of their genes (Aquea et al. 2010). Another study, using a similar approach to the previous, identified not only HATs and HDACs but also HMTs and HDMTs, relating their expression patterns to various biological processes (Wang et al. 2020).

The third pillar of epigenetics is the regulation by ncRNAs, of which the most relevant for epigenetic processes are siRNA and lncRNA. Small interfering RNAs can originate from both endogenous and exogenous sources and can play roles in both PTGS and RdDM, depending on whether they are loaded in AGO1 or AGO4/6, respectively (Sanan‐Mishra et al. 2021; Waheed et al. 2021). Endogenous siRNA can also emerge from the canonical RdDM pathway, resulting from DICER‐LIKE3‐mediated cleavage of dsRNAs transcribed from RNA Pol IV, or from the cleavage of miRNA precursors (Erdmann and Picard 2020). Some long non‐coding RNA, on the other hand, can interact with and modulate chromatin (reviewed in Chen et al. 2021). Beyond epigenetic regulation, other ncRNAs also play important roles in gene expression regulation, a quintessential example being micro RNAs in the post‐transcriptional gene silencing (PTGS) pathway (Yang et al. 2021). Although not epigenetic in nature, such examples will be discussed alongside other cases.

## 3 EPIGENETICS AND PLANT IMMUNITY – ANOTHER LAYER OF REGULATION

From the model plant Arabidopsis to crop plants, the role of epigenetic regulation plays in plant immunity is being unraveled at an increasingly faster pace. DNA methylation is among the better‐studied epigenetic mechanisms in this context, with hypomethylation appearing to be important in improved immune responses (Geng et al. 2019; Lee et al. 2023), although the opposite has also been observed for necrotrophic pathogens (López Sánchez et al. 2016). This hypomethylation may also be actively imposed, as evidenced by the fact that mutants lacking DNA demethylases are left more susceptible to pathogens (López Sánchez et al. 2016; Zeng et al. 2021; Huang et al. 2022; Zhang et al. 2022). The general line of reasoning follows that hypomethylation is behind the transcriptional activation. This remodeling is also strongly linked to TEs, since their DNA methylation patterns are known to be modulated by stress. Indeed, the case of *TNL RESISTANCE METYLATION GENE* 1 (*RMG1*) is representative of this case, where it was shown that ROS1 actively demethylates a TE embedded in its promoter upon infection. This leads to the derepression of *RMG1*, contributing to resistance against *Pseudomonas syringae* pv. *tomato* (Halter et al. 2021). Despite this form of proximal regulation of DNA methylation, termed *cis*‐regulation, a considerable amount of evidence also points towards these patterns governing the expression of distal genes (i.e., *trans*‐regulation) (Hannan Parker et al. 2022). This form of regulation is also reliant on TEs, but this case is mediated by siRNAs that are generated from TE cleavage during their re‐silencing after an initial infection. Besides targeting TEs for RdDM, these siRNAs are also involved in promoting defense gene expression, with this mechanism being hypothesized to be mediated by AGO1 H2A.Z in gene coding regions (Cambiagno et al. 2018; Liu et al. 2018; Hannan Parker et al. 2022).

Histone tail modifications have too been implicated in the plant immune response: from acetylation acting as a key regulator of JA signaling (Zhou et al. 2005; Vincent et al. 2022) to methylation playing a role in the JA‐salicylic acid (SA) crosstalk (Alvarez‐Venegas et al. 2007). In addition to histone modifications *per se*, other chromatin remodelers play important roles in the plant immune response, being frequently linked to transcriptional regulation of NLR genes. This has already been reported in Arabidopsis, for example, for Chromatin remodeler 5 (CHR5) (Zou et al. 2017), Splayed (SPY) (Johnson et al. 2015), and Switch/sucrose non‐fermentable (SWI/SNF)‐associated protein (SWP73A) (Huang et al. 2021), the latter two being constituents of the SWI/SNF complex, evidencing the importance of chromatin remodeling in the establishment of ETI.

Despite the importance of chromatin changes that take place throughout the infection course, what arises after this *rendezvous* is also of significance. Indeed, plants are able to hold a molecular memory after infection, which produces stable changes from the epigenetic and transcriptional to metabolic levels, culminating in an induced resistance phenotype (Harris et al. 2023). One quintessential example of priming against pathogens is found in systemic acquired resistance (SAR), where the plant develops a systemic response after a local infection, and is able to hold a memory at the somatic level in the short‐term (Fu and Dong 2013). Incidentally, epigenetics has long been hypothesized to play a role in SAR establishment, with some (albeit limited) evidence even pointing towards a possible transgenerational transmission of this state (Luna and Ton 2012; Luna et al. 2012). At the (epi)genetic level, TEs play once again a central role in the establishment of priming states. Indeed, it has been hypothesized that miRNAs and siRNAs produced from TEs, which are stress‐activated, can remain bound to AGO1 after stress. The AGO1‐sRNA complex then accumulates at the promoters of defense genes and recruits RNA Polymerase II, although it is kept paused by H2A.Z, which is deposited after the stress dwindles. Because of this, when a subsequent stress is imposed, primed defense genes are more quickly activated, thus establishing a sensitized state at the genetic level (Hannan Parker et al. 2022).

However, priming can also be performed without exposing plants to stress, since treating plants with priming molecules such as PAMPs, specific hormones or other related signaling molecules can produce similarly effective results. In fact, both DNA methylation and histone modifications have been implicated in memory establishment, both somatically and transgenerationally, after pathogen challenge or chemical elicitation (Harris et al. 2023).

As it stands, knowledge regarding epigenetic processes in plant‐pathogen interactions is quickly compounding, although more focused on chromatin dynamics that take place during the infection course. However, understanding the memory component of epigenetics is essential, not only at a fundamental level but also within the context of plant immunity (i.e., priming), something that is currently lacking. In Harris et al. (2023), the authors outline key questions such as the lack of knowledge around causality of epigenetic marks and priming, as well as how specific the induced changes are, depending on which stress is being applied. Alongside the stability of these marks after priming, these amount to the largest questions when it comes to epigenetic stress memory. Answering these questions will surely lead to a better understanding of this complex regulatory mechanism and will also enable researchers to harness it for future biotechnological applications.

## 4 EPIGENETIC REGULATION IN GRAPEVINE IMMUNITY

Although a relatively new study topic in the field of grapevine research, epigenetic regulation of the immune response has recently garnered increased attention from all facets (summarized in Table 1).

**TABLE 1:** Summary of current research in grapevine‐pathogen interactions, from the scope of epigenetic regulation and non‐epigenetic, non‐coding RNAs.

Pathogen	Studied regulatory mechanism	Epigenetic marks directly assessed?	Biological effect	Reference
**Epigenetic mechanisms**				
*P. viticola*	DNA methylation	Yes, but global methylation only	Lower global methylation levels in tolerant genotypes in response to infection. Reprogramming of DNA methylation‐ and chromatin remodeling‐associated genes.	(Azevedo et al. 2022; Pereira et al. 2022)
Candidatus *P. vitis*	DNA methylation	Yes	Differential methylation patterns in two‐year recovered vs. healthy plants.	(Pagliarani et al. 2020)
*B. cinerea*	DNA methylation	Yes	Melatonin elicitation reprograms methylation in promoters of immunity‐related genes and increases tolerance to *B. cinerea*	(Gao et al. 2020)
GLRaV‐3	Histone acetylation	No	Early upregulation of HAT genes in response to viral infection	(Aquea et al. 2010)
**Non‐epigenetic mechanisms**				
*P. viticola*	phasiRNA, siRNA	N/A	Various grapevine‐derived sRNAs target *P. viticola* transcripts, and vice‐versa, including siRNA, phasiRNA, and miRNA	(Brilli et al. 2018)
*L. theobromae*	lncRNA	N/A	Differential accumulation of lncRNAs between tolerant and susceptible, targeting immunity‐related genes	(Xing et al. 2019)
*P. viticola*	lncRNA	N/A	Grapevine lncRNA coexpresses with genes linked to JA and chalcone biosynthesis, genes encoding PR proteins and WRKY TFs.	(Bhatia et al. 2021)
*E. necator*	lncRNA	N/A	Grapevine lncRNA coexpresses with genes linked to SA and cell wal degradation, genes encoding PR proteins and WRKY TFs.	
*B. cinerea*	lncRNA	N/A	Co‐expression of pathogen‐responsive lncRNA targeting genes associated with stilbenoid biosynthesis, chitin degradation. Potential decoys for miRNAs targeting PR protein‐encoding genes	(Bhatia et al. 2020)

The role of DNA methylation, for instance, has been highlighted in the context of grapevine downy mildew (DM), caused by the obligate biotroph oomycete *Plasmopara viticola*. It was shown that genotypes tend to present lower levels of DNA methylation compared to their susceptible counterparts (Azevedo et al. 2022; Pereira et al. 2022). Furthermore, differences at the transcriptional level were also uncovered, with genotypes carrying a Rpv3 background presenting a stronger downregulation of DNA methyltransferase gene expression during infection (Pereira et al. 2022). This is in line with the current understanding of the role of DNA methylation in plant‐pathogen interactions, where hypomethylation is generally linked to transcriptional activation and, subsequently, to an improved immune response (Zhang et al. 2018; Tirnaz and Batley 2019). Furthermore, this suggests that grapevine tolerance to *P. viticola* may also be explained at the epigenetic level. Nevertheless, more detailed knowledge regarding this is much needed since global methylation is not informative enough for conclusions to be drawn. Questions such as (1) which genes or other genomic regions are differentially methylated, (2) if and how different are these regions between different genotypes, or (3) if these patterns are maintained after the infection are central regarding the role of epigenetics in the plant immune response.

When it comes to long‐lasting epigenetic changes in response to pathogens, only one study has explored the importance of epigenetics, DNA methylation in particular, in two‐year recovered grapevine plants previously infected with *Candidatus Phytoplasma vitis*, the causal agent of *Flavescence dorée* (FD) (Pagliarani et al. 2020). In this study, methylation landscapes were first noted to be distinct between healthy and two‐year recovered plants, with most of the difference being present at the gene body level when compared to promoters. Simultaneously, it was found that genes related to sugar and flavonoid metabolism were downregulated in recovered plants compared to healthy ones, along with photosynthesis‐related genes. Many of these genes also showed hypermethylation, indicating a possible role for DNA methylation in transcriptome remodeling post‐infection, and suggesting a memory can last up to two years after FD onset. However, true priming effects were not evaluated since no re‐infection was performed. Understanding if the response of these plants to re‐infection was improved would be crucial in better understanding the mechanisms of priming against disease in grapevine and in ascribing a role for DNA methylation in this process.

Grapevine has also received considerable attention regarding priming with elicitors against pathogens, with the epigenetic component having recently been implicated in priming with melatonin. Indeed, Gao and associates (2020) found that melatonin treatment improved the response against *Botrytis cinerea*, while inducing hypomethylation at the promoter level of defense‐related genes, correlating with their upregulation. Like the previously mentioned work concerning FD, one cannot conclude true priming effects arise from melatonin since no short‐ or long‐term memory was tested. Nevertheless, the observed transcriptional and methylome reprogramming events are noteworthy, showing the *cis*‐regulation of defense genes in response to elicitation, and provide a basis for further work in this crop plant. It would be of relevance to assess actual priming responses, which could be approached by temporally spacing infection events or infection from elicitation, evaluating the short‐term somatic memory these marks possess.

As for the role of histone modifications in grapevine immunity, none of the more commonly studied marks have been directly studied, with the only clues consisting of gene expression of histone‐modifying enzyme‐encoding genes. For instance, grapevine leafroll‐associated virus 3 (GLRaV‐3) infection induced changes in the expression of HAT genes (Aquea et al., 2011a), while infection with *Erysiphe necator*, the causal agent of powdery mildew (PM), also triggered upregulation of several genes of this family 12 hours after infection, including *VvHAC1*, *VvHAG4* and *VvHAG23* (Wang et al. 2020). Histone acetylation is generally linked to euchromatic regions and expressed genes (Shahbazian and Grunstein 2007; Maeshima et al. 2024), while deacetylation was noted to negatively affect immunity upon pathogen challenge in Arabidopsis (Yang et al. 2020) and rice (Ding et al. 2012). Histone acetylation could therefore be a possible mechanism involved in transcriptional activation upon infection in grapevine, although more pathosystems must be studied and the epigenetic states directly assessed. Indeed, no other works aimed to characterize histone‐modifying enzymes in the context of grapevine‐pathogen interactions, and no studies exist whatsoever where specific histone modifications are directly analyzed, representing a glaring gap in epigenetic research in this specific context.

As to the role of ncRNAs in grapevine immunity, the distinction between epigenetic and non‐epigenetic mechanisms is once again worth noting. For instance, while miRNAs are generally not epigenetic, rather than being an integral part of PTGS, siRNA and lncRNA can be epigenetic (see section Mechanisms of epigenetic regulation). Currently, studies relating the role of siRNA with chromatin dynamics in grapevine are next to non‐existent. Indeed, up to our knowledge, there is only one work where siRNAs are linked to DNA methylation modulation via *de novo* methylation, performed in grapevine embryogenic *calli*. In it, it was shown that CHH hypermethylation arises in heterochromatin of grapevine *calli*, which was attributed to *de novo* methylation driven by heterochromatic siRNA that concomitantly accumulated in this tissue (Lizamore et al. 2021). This could also represent an interesting approach for introducing novel epigenetic regulation during tissue culture, something that could be harnessed for future breeding programs.

Beyond this, the only work with ncRNAs in the field of grapevine immunity is restricted to post‐transcriptional control of gene expression, representing a glaring gap in the knowledge of the role of these molecules in epigenetic processes. Nevertheless, they represent another important layer of gene expression regulation.

Bioinformatic prediction of trans‐acting siRNAs (tasiRNA) encoded in the *VvTAS7* locus points towards the regulation of several genes that may be linked to immune responses, including a leucine‐rich receptor‐like protein kinase, a histone acetyltransferase, and a fatty acid desaturase (Zhang et al. 2012). All these genes have been linked to plant immunity against many taxa, in grapevine or otherwise, which provides clues for the role of these tasiRNAs in this same context (Couto and Zipfel 2016; Ramirez‐Prado et al. 2018; Laureano et al. 2021). Another interesting role of tasiRNAs (and all sRNAs alike) is their role in host‐pathogen crosstalk. It has been found that *VvTAS3* encodes siRNAs that specifically target *P. viticola* transcripts, which may indicate that these sRNAs may act not only within the plant, but also as a direct mechanism of immunity. Likewise, *P. viticola*‐derived sRNAs targeting grapevine transcripts have also been uncovered, one of which is putatively targeting an LRR‐like protein kinase, an important family of proteins in plant immunity, while grapevine‐derived sRNAs originate mostly from disease‐related genes (Brilli et al. 2018). A scenario in which cross‐kingdom sRNA crosstalk can alter the fate of grapevine‐pathogen interactions opens new ways to tackle this problem since novel endogenous plant sRNAs could be engineered to target specific pathogen genes, hindering their pathogenicity.

Besides sRNAs, novel evidence has shown that lncRNAs may likely play a role in grapevine immunity against various pathogens. One study explored lncRNA profiles upon infection by *Lasiodiploidia theobromae*, where it was possible to discriminate the tolerant cultivar ‘Merlot’ from the susceptible ‘Cabernet Franc’ based on lncRNA profile alone. Additionally, many identified lncRNAs were positively correlated with the expression of genes related to chitin catabolism and cell wall dynamics, which have been shown to be of importance in plant‐fungal interactions (Xing et al. 2019). Although the authors did not explore this regulatory role further, this is of particular interest in the scope of epigenetic regulation, given the role some lncRNAs have in chromatin remodeling. Recently, it was found that the Arabidopsis lncRNA *salicylic acid biogenesis controller 1 (SABC1)* acted as a negative regulator of SA‐mediated immune responses by silencing a NAC transcription factor, *NAC3*, that promotes SA synthesis. This repression was due to the recruitment of the PRC2 to the locus of NAC3, which resulted in the deposition of the repressive H3K27me3 mark. Interestingly, SABC1 is repressed during infection, releasing the repression of *NAC3* and allowing SA accumulation (Liu et al. 2022). It is thus possible that lncRNAs correlated with transcriptional activation of other genes may imply that chromatin remodeling can occur, although this would require a finer analysis of the systems at hand. A similar case emerged in the interactions with *P. viticola*, *E. necator* and *B. cinerea*, where many functionally related genes appeared positively regulated to lncRNAs modulated by the infection with these pathogens. For example, *P. viticola* infection was shown to increase lncRNAs correlated to JA signaling‐associated genes, while *E. necator* triggered the ones correlated to SA signaling (Bhatia et al. 2021), two phytohormones that play key roles in the interactions with these pathogens (Weng et al. 2014; Guerreiro et al. 2016). Conversely, in the interaction with *B. cinerea*, a putative association to processes such as chitin degradation or stilbenoid biosynthesis was attributed to some lncRNA molecules (Bhatia et al. 2020). Interestingly, both works uncovered a potential role for pathogen‐responsive lncRNAs as decoys for miRNAs implicated in PTGS of defense‐related genes. Indeed, DM‐ and PM‐responsive lncRNAs may bind miRNAs targeting MYB transcription factors, which are important molecular players in immune responses (Yu et al. 2023). Interestingly, PM, DM, and *B. cinerea* infections triggered the accumulation of a lncRNA found to target vvi‐miR482, a miRNA associated with NLR gene silencing. These findings suggest an important role of lncRNAs in regulating PTGS in response to pathogens, and suggests a conserved function in grapevine immune responses (Bhatia et al. 2020, 2021).

## 5 EPIBREEDING, A NEW FRONTIER IN GRAPEVINE BREEDING

**FIGURE 2:**
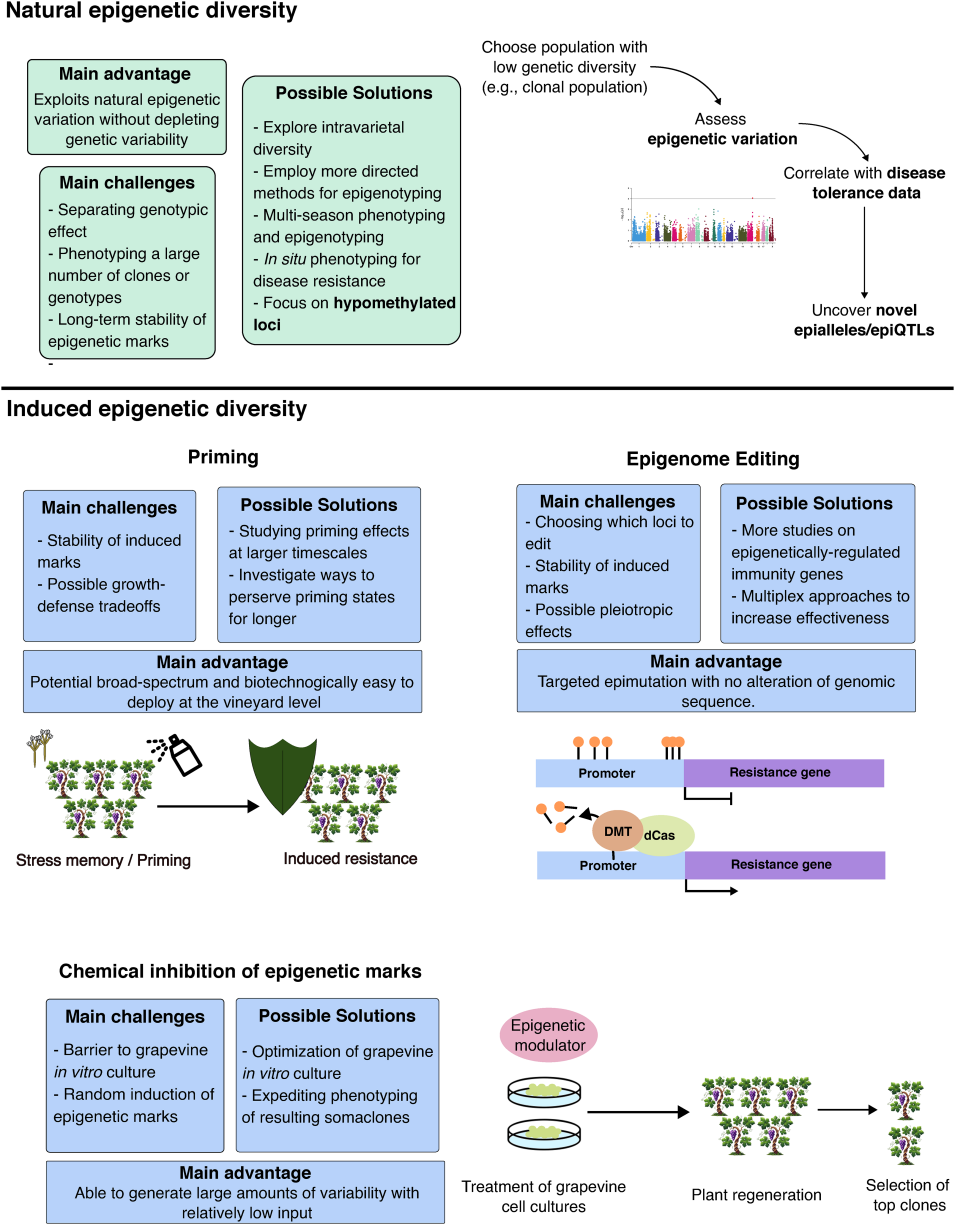
Possible paths of introducing epigenetic marks in grapevine breeding, from harnessing natural epigenetic diversity (top panel) to inducing it (bottom panel). epiQTL – epigenetic quantitative trait locus; DMT – DNA demethylase; dCas – dead Cas.

Although in‐depth knowledge of epigenetic regulation of grapevine immunity remains elusive, there are now robust clues that implicate these mechanisms in this ever‐important aspect of grapevine biology. Accordingly, it has become particularly enticing to speculate on how these mechanisms could be employed in grapevine breeding (termed epibreeding), with this exercise already having been done very recently for environmental changes in general (Berger et al. 2023; Tan and Rodríguez López 2023) and to other aspects of grapevine biology (Venios et al. 2024). Although modern breeding techniques, which rely on the deployment of resistance genes, can be extremely effective, having another layer of regulation represents another tool in the arsenal of breeders to improve resilience. Epibreeding can also be valuable in preserving genetic landscapes and their variability, as it may not require genome editing. However, it is important to understand not only the possible benefits of epibreeding in the scope of improving grapevine resilience to disease but also its limitations in the agronomical context it exists in. Therefore, in this section, we aim to pose new perspectives on epibreeding applied to grapevine immune responses while critically analyzing the feasibility of different strategies (summarized in Figure 2).

As it stands, there are two possible ways to approach epibreeding: 1) to explore naturally occurring epigenetic variability; and 2) to induce new epigenetic marks. Exploring natural variability is generally associated with epigenome‐wide association studies (EWAS), the discovery of epigenetic quantitative trait loci (epiQTL) or epialleles – in essence, the correlative study of preexisting epigenetic marks that are widespread in populations and a given phenotype.

Very recently, a naturally occurring, heritable epiallele regulating the expression of two adjacent NLR genes was uncovered and was linked to heightened clubroot disease tolerance in Arabidopsis (Gravot et al. 2024). The existence of naturally occurring, epigenetically regulated expression polymorphisms of defense‐related genes sets a precedent for investigating epigenetic variability in other populations, including crop plants.In grapevine, natural epigenetic variability has been a subject of study, and is a possible factor behind the observed phenotypic plasticity (Xie et al. 2017; Varela et al. 2021). Furthermore it has been demonstrated that environmentally‐induced DNA methylation marks are not only maintained up to three years after transplantation (Varela et al. 2024), but also in plants that recovered from FD (Pagliarani et al. 2020). With these aspects in mind, it becomes enticing to enquire if there are naturally occurring epigenetic marks associated with increased tolerance to diseases in grapevine, how they exert their biological role and how stable they are on a multi‐year scale. To approach this in a robust manner, it is essential to remove as much of the genotypic effect as possible, which is particularly relevant in grapevine due to its high intervarietal genetic diversity. One way to possibly overcome this is to study grapevine at the intravarietal level (i.e., different clones of the same genotype), which also displays significant phenotypic diversity that arose due to longstanding practices of vegetative propagation (Gonçalves and Martins 2022). This diversity has been harnessed in the past in breeding programs for viticultural and oenological traits (Gonçalves and Martins 2022), as well as for drought stress tolerance (Carvalho et al. 2020), but never in the scope of increased tolerance to disease. Although it is known that clonal diversity can be linked to epigenetics (Guarino et al. 2015), including grapevine (Varela et al. 2024), its effect on phenotypic diversity was never assessed in the scope of immunity or any other type of stress. For this, directing efforts towards extensive epi‐genotyping and phenotyping in consecutive seasons is key, with particular care towards the environmental context in which these are done, as well as the genetic background of the studied populations. For instance, susceptibility assays in grapevine are often performed in leaf disk, and the inoculation is performed under tightly controlled conditions. If the point of these efforts is to uncover epigenetic variation among individuals, it is possible that this procedure may introduce biases in these same mechanisms, possibly leading to confounding effects.

Exploring the naturally occurring heritable epigenetic changes is, however, not the last frontier of epibreeding since these changes can be artificially induced. Some strategies, ranging from the development of epigenetic recombinant inbred lines (epiRIL) or the induction of novel marks using epigenetic inhibitors (e.g., DNA methylation or HDAC inhibitors) to more cutting‐edge methods of epigenome editing, could be employed for epibreeding in grapevine.

A promising new technique for inducing changes to the epigenome in a site‐specific can be found in epigenome editing. These strategies generally rely on harnessing dead Cas (dCas), a mutated Cas protein without nuclease activity, in fusion with an epigenetic writer or eraser to direct the modulation of epigenetic marks to specific loci (Gardiner et al. 2022). This is especially powerful if the knockout or overexpression of genes is not adequate for a crop system since these can have pleiotropic effects and hinder other important agronomical traits (Derbyshire et al. 2024). Rather, epigenome editing can act as a dial for gene expression, rather than the binary effect (i.e., overexpression and knockout) that is found in traditional genome engineering approaches. For instance, the silencing of a sucrose transporter in cassava, whose promoter was known to be unmethylated, via targeted DNA methylation was sufficient to improve resilience to bacterial blight (Veley et al. 2023).

The use of epigenome editing is, of course, dependent on the knowledge of immune‐related genes that are under epigenetic control which, in grapevine, is still limited. As an initial approach, it is essential to uncover immune‐related genes that are under control of chromatin mechanisms and if these are maintained under epigenetic memory. Although both cases have separately been achieved, providing some evidence to this end, a unified vision of epigenetically regulated immunity genes is still required. Epigenome editing also suffers from the same hindrance of transformation, although no crossing is required, making it a more feasible alternative to EpiRILs. It is also important to note that, although difficult, a lot of progress has been made regarding grapevine transformation and regeneration, including boosting resilience to disease (Moffa et al. 2024). Additionally, the advent of the New Genomic Techniques (NGT) has paved the way for increased acceptance of genetic engineering in crop species, although the legal framework is yet to be set. Once more knowledge on which marks are epigenetically regulated in the context of immune responses, novel targets can be elected for these approaches.

Conversely, the induction of new epigenetic marks without resorting to genome editing techniques, although perhaps less targeted, may prove more fruitful for future breeding efforts. For instance, the use of epigenetic inhibitors could prove to be more feasible, since it only requires *in vitro* culture of grapevine and their treatment with said inhibitors. Indeed, this technique has already been reported in grapevine somaclones treated with DNA methylation inhibitors, although the clones are still awaiting phenotyping (Berger et al. 2023). This strategy is particularly enticing because it generates a considerable amount of epigenetic variability, albeit randomly, that could be harnessed for various aspects of grapevine biology: from development to stress tolerance. Furthermore, inducing DNA hypomethylation is made even more interesting in the context of grapevine‐pathogen interactions, having been previously reported to be associated with tolerance (Azevedo et al. 2022; Pereira et al. 2022), in line with the scientific consensus. Nevertheless, the barrier of grapevine *in vitro* culture is worth bearing in mind, especially when considering its general recalcitrance due to high genotype and explant specificity (Nuzzo et al. 2022).

Another promising facet of induction of epigenetic reprogramming lies in immune priming. Although priming is not a novelty for the potentiation of plant immunity, the notion of epigenetics playing a major role in transcriptional reprogramming and memory maintenance has been gaining traction. Indeed, both DNA methylation and chromatin‐associated mechanisms, such as nucleosome occupancy or histone modifications, have been linked to priming memory, with specific marks being associated with different types of memory (Harris et al. 2023). In grapevine, various immune priming methods have been studied, from chemical priming agents such as laminarin (Gauthier et al. 2014), fatty acids (Laureano et al. 2024) or lipopolysaccharides (Castro et al. 2023), to biopriming (i.e., priming with beneficial microbes) (Jeandet et al. 2023; Langa‐Lomba et al. 2023). Often, these works examine not only the immediate effects of priming but also the short‐term memory modulation that takes place, generally at the transcriptional level. However, there are currently no studies that examine if and how epigenetic marks are modulated, their role in transcriptional reprogramming and their stability in the short‐, medium‐ and long‐term.

## 6 CONCLUDING REMARKS AND FUTURE PERSPECTIVES

Although much is yet to be understood, it has become clear that epigenetic marks and all their facets play a key role in plant immune responses, from gene expression regulation to memory. Moreover, some non‐epigenetic players, as some lncRNAs, have also been shown to be important to the outcome of plant‐pathogen interactions. Most of this knowledge is, however, still circumscribed to the model plant Arabidopsis, while mechanisms in crop plants are significantly less elucidated, grapevine being no exception.

Recently, some progress has been made regarding these very mechanisms in grapevine. Differential responses at the DNA methylation level in a genotype‐dependent manner were described, along with gene expression regulation of other writers, erasers and readers of chromatin, in response to downy mildew. Elicitation was also found to reprogram DNA methylation at the promoters of immunity‐regulated genes, and strong clues for epigenetic memory were uncovered in plants recovered from FD. Albeit limited, this provides a robust foundation for pursuing further research in this crop plant, which we propose should be directed towards two key points:

1. Understand the responsiveness of epigenetic marks to infection and their stability in its aftermath

Are there disease‐responsive genes that are controlled by epigenetic mechanisms, and is this control genotype‐dependent? The coupling between genetic and epigenetic components has been previously described in other models (Taudt et al. 2016) and reconciling this with the differential response between genotypes suggests the same could occur in the grapevine immune response. It is also enticing to investigate the stability of these marks, distinguishing them from chromatin regulation of gene expression.

2 Explore epigenetic diversity in inter‐ and intra‐varietal diversity

Are there stable epigenetic marks that confer increased tolerance to disease in grapevine? Could those explain part of the observed phenotypic diversity among clones or even varieties? Searching for natural epigenetic variation in grapevine populations could hold tremendous potential in the scope of plant immunity, but it is not without its difficulties. First, it is important to establish if grapevine intravarietal diversity observed in other important traits also extends to susceptibility to disease, requiring robust phenotyping of selected varieties. By looking at this level, genetic variation is minimized and solely the epigenetic effect can be assessed. Nevertheless, it could also be of interest to assess if epigenetics also plays a role in intervarietal diversity, possibly interplaying with the already well‐known genetic diversity. Novel methods, such as the one developed by Lesur and associates (2024), enable the study of epigenetics at the population level, considering naturally occurring genetic variations and reconciling strong epigenomic characterization with a reduced operational cost. This approach could also be applied to grapevine populations, enabling higher resolution analyses, which have relied on low‐throughput techniques like MSAP in the past (Varela et al. 2024).

## AUTHOR CONTRIBUTIONS

JPP, IB and SV wrote the manuscript. JPP prepared the figures. All authors have read and approved the manuscript.

## FUNDING INFORMATION

This work was supported by the European Commission in the frame of the Horizon Europe program, project ‘Shield4Grape’ (grant agreement number 101135088), and by UIDB/04046/2020 (doi: 10.54499/UIDB/04046/2020) and UIDP/04046/2020 (doi: 10.54499/UIDP/04046/2020) Centre grants the Portuguese Foundation for Science and Technology (FCT, Portugal) to BioISI. RBS was funded by FCT through the CEEC program (doi: 10.54499/2021.00795.CEECIND/CP1654/CT0005). JPP was funded through the FCT PhD Studentship (2024.00649.BD).

## Data Availability

Non‐applicable.
